# A Neuroimmune Modulator for Alcohol Use Disorder: A Randomized Clinical Trial

**DOI:** 10.1001/jamanetworkopen.2025.7523

**Published:** 2025-04-30

**Authors:** Lara A. Ray, Lindsay R. Meredith, Erica N. Grodin, Malia A. Belnap, Steven J. Nieto, Wave A. Baskerville, Suzanna Donato, Steven J. Shoptaw, Artha J. Gillis, Michael R. Irwin, Karen Miotto, Craig K. Enders

**Affiliations:** 1 Department of Psychology, University of California at Los Angeles; 2 Department of Psychiatry and Biobehavioral Sciences, University of California at Los Angeles; 3 Brain Research Institute, University of California at Los Angeles; 4 Center for Behavioral and Addiction Medicine, Department of Family Medicine, University of California at Los Angeles

## Abstract

**Importance:**

The neuroimmune system represents a promising target for novel medications for alcohol use disorder (AUD). Ibudilast is a neuroimmune modulator which selectively inhibits phosphodiesterases (PDE) 3, PDE4, PDE10, and PDE11, and macrophage migration inhibitory factor (MIF).

**Objective:**

To test the efficacy of ibudilast for AUD compared with placebo.

**Design, Setting, and Participants:**

This randomized clinical trial was a double-masked, phase 2 trial conducted at an academic research center between October 2018 and April 2023. Eligible participants were adults seeking treatment for moderate or severe AUD. After completing the 12-week treatment period, participants were followed up for an additional 4 weeks.

**Interventions:**

Ibudilast taken twice daily in 50 mg doses for 12 weeks vs placebo.

**Main Outcomes and Measures:**

Percentage of heavy drinking days; secondary efficacy outcomes included drinks per day, drinks per drinking day, and percentage of days abstinent. Registered exploratory analyses tested whether the effects of ibudilast of drinking outcomes were moderated by baseline depressive symptomatology. Registered exploratory analyses also tested whether ibudilast reduced inflammation compared with placebo, as indicated by circulating levels of proinflammatory markers over the 12-week trial. Additionally, a post hoc exploratory analysis investigated whether sex moderated the effect of ibudilast on drinking outcomes. Data were analyzed between October 2018 and April 2023.

**Results:**

A total of 102 participants were enrolled in the study (mean [SD] age, 44.3 [10.8] years; 61 male [59.8%]; 24 Black [23.5%], 32 Hispanic [31.4%], 52 White [51.0%]). Baseline demographic characteristics and alcohol use patterns did not significantly differ between the 2 medication conditions. There was no significant difference between ibudilast vs placebo on percentage of heavy drinking days (β = 0.06, SE = 0.08 [95% CI, −0.09 to 0.21]; *P* = .46). There were no significant differences between ibudilast vs placebo on registered secondary outcomes. There were no significant effects of ibudilast compared with placebo on peripheral markers of inflammation. Moderation analyses found that baseline depressive symptomology (time 2 for drinks per drinking day: β = 0.25, SE = 0.11 [95% CI, 0.03 to 0.48]; *P* = .03) and sex (β = −2.48, SE = 1.07 [95% CI, −4.59 to −0.37]; *P* = .02) may moderate the effects of ibudilast.

**Conclusions and Relevance:**

In this randomized clinical trial of ibudilast for the treatment of AUD, there was no support for the efficacy of ibudilast over placebo; additionally, no effect of ibudilast on markers of peripheral inflammation was observed. As novel treatments for AUD are developed for novel molecular targets, their effects may depend on mechanisms and moderators of efficacy.

**Trial Registration:**

ClinicalTrials.gov Identifier: NCT03594435

## Key Points

**Question:**

Does ibudilast reduce the percentage of heavy drinking days compared with placebo over a 12-week treatment period in individuals with alcohol use disorder?

**Findings:**

In this randomized clinical trial of 102 treatment-seeking adults with moderate to severe alcohol use disorder, a twice-daily 50 mg dose of ibudilast did not significantly reduce the percentage of heavy drinking days compared with placebo.

**Meaning:**

These findings do not support the efficacy of ibudilast for treating alcohol use disorder.

## Teaser

This randomized clinical trial examines the effectiveness of ibudilast, a neuroimmune modulator, to treat alcohol use disorder.

## Introduction

A mounting body of research suggests that the immune system plays a vital role in alcohol use disorder (AUD).^1,2^ Chronic, heavy alcohol intake alters immune signaling and increases neuroinflammation—whereby alcohol indirectly initiates systemic production of proinflammatory cytokines (promoting inflammatory processes) and directly stimulates the release of inflammatory molecules in the brain, all of which induces neural damage.^3,4^ Additionally, systemic inflammation may be provoked by alcohol when it acts on peripheral immune receptors in the gut,^5^ permitting inflammatory molecules to leak into the bloodstream, a phenomenon known as “leaky gut.”^6^ A prolonged inflammatory response, such as one stemming from sustained heavy alcohol use, can contribute to psychiatric and physical disorders.^7^ There is a robust interplay between the immune system and AUD,^8^ and as such, the neuroimmune system represents a promising novel target for medications for AUD.^9^

Ibudilast (MN-166, previously AV411) has been used in Japan for asthma and cerebrovascular disorders.^10^ Ibudilast preferentially inhibits phosphodiesterase 3A (PDE3A), PDE4, PDE10A, and PDE11A and inhibits macrophage migration inhibitory factor (MMIF).^11^ As PDE4 and MMIF are critically involved in proinflammatory signaling,^12,13^ and PDE10 negatively regulates neurotrophin expression,^14^ the inhibition of these molecules by ibudilast is thought to reduce neuroinflammation and promote neurotrophin expression.^11^ In support, ibudilast enhances neurotrophin expression, reduces proinflammatory cytokine release, and attenuates neuronal death in vitro.^15^ In animals, ibudilast reduced ethanol intake by approximately 50% during maintenance and relapse testing.^16^ These results advanced ibudilast as a promising treatment for AUD.

Toward developing ibudilast for AUD, our laboratory previously conducted 2 independent clinical studies. The first trial found that ibudilast decreased tonic craving for alcohol and improved mood following alcohol cue and stress exposure, as compared with placebo.^17^ The second trial found that ibudilast reduced rates of heavy drinking and neural alcohol cue-reactivity over a 2-week treatment period.^18^ In addition, participants treated with ibudilast had lower choline-containing compounds in superior frontal white matter and lower myo-inositol in pregenual anterior cingulate cortex, neurometabolites reflective of decreases in neuroinflammation.^19,20^ Secondary analysis suggested that individuals with elevated CRP levels at baseline had the most favorable clinical response to ibudilast.^21^

To translate these promising early efficacy findings, this study consists of a double-masked, randomized, single-site trial testing the efficacy of ibudilast (50 mg/twice daily), compared with placebo, on alcohol use over a 12-week treatment period. The primary outcome was percentage of heavy drinking days (PHDD) and we hypothesized that ibudilast treatment would be associated with lower PHDD compared with placebo. Registered secondary outcomes were drinks per day, drinks per drinking day, and percentage of days abstinent. Preregistered exploratory analyses examined the moderating role of baseline depressive symptomology and tested whether ibudilast, compared with placebo, reduced peripheral inflammation over the 12-week trial. Post hoc exploratory analyses evaluated biological sex as a moderator.

## Methods

### Trial Design

**Figure 1.**
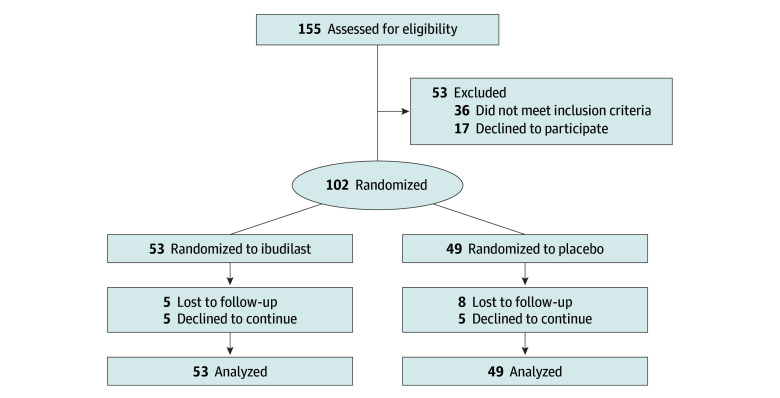
CONSORT Flow Diagram

This study was a phase 2, 12-week, double-masked, placebo-controlled randomized clinical trial of ibudilast (50 mg twice daily) for treating AUD (ClinicalTrials.gov identifier: NCT03594435), randomizing participants who were treatment-seeking with current AUD (Figure 1). Participants completed telephone screening, an in-person eligibility assessment, a medical physical examination, randomization to study medication or matched placebo, and in-person follow-up visits at 4, 8, and 12 weeks. During the COVID-19 pandemic, several in-person follow-up visits were converted to telephone or video-based assessments. Forty participants completed the trial prior to start of the COVID-19 pandemic, and 62 participants completed after the start of the pandemic. For participants enrolled during the early stages of the COVID-19 pandemic, assessments of alcohol use at weeks 2, 6, and 10 were administered via telephone.

This trial was approved by the University of California, Los Angeles institutional review board and monitored by a data and safety monitoring board. All study participants provided written informed consent. Participants were enrolled and followed in the study between October 2018 and April 2023. Data analysis was conducted from February through July 2024. This report adheres to the Consolidated Standards of Reporting Trials (CONSORT) reporting guidelines.

### Setting and Participants

The trial was conducted at an outpatient research facility at the University of California, Los Angeles (UCLA) and at the Westwood/UCLA Clinical Translational Research Center (CTRC). Participants were recruited through print, social media, and mass transit advertisements. Included participants were: (1) aged between 18 and 65 years; (2) met the *Diagnostic and Statistical Manual of Mental Disorders* (Fifth Edition) (*DSM-5*) diagnostic criteria for moderate or severe alcohol use disorder in the past 12 months; (3) sought treatment for AUD; and (4) reported drinking at least 14 drinks per week for male participants (7 drinks per week for female participants) in the 28 days prior to consent.

The following were exclusion criteria: (1) a *DSM-5* diagnosis of substance use disorder other than alcohol and nicotine in the last 12 months (mild cannabis use disorder was allowed); (2) a lifetime *DSM-5* diagnosis of schizophrenia, bipolar disorder, or any psychotic disorder; (3) a positive urine screen for opioids, amphetamines, or sedative hypnotics; (4) clinically significant alcohol withdrawal symptoms, indicated by a score 10 or higher on the Clinical Institute Withdrawal Assessment for Alcohol-Revised (CIWA-Ar)^22^; (5) pregnancy, nursing, or not using reliable birth control (if female); (6) medical condition that could interfere with safe study participation, such as unstable cardiac, kidney, or liver disease; (7) aspartate aminotransferase, alanine aminotransferase, or γ-glutamyl transpeptidase levels 3 times the upper normal limit or higher; (8) an attempted suicide in the past 3 years or serious suicidal intention or plan in the past year; (9) using prescription medication contraindicated with ibudilast, including alpha or beta agonists and theophylline; (10) current use of any psychotropic medications, except antidepressants at stable dose for 4 or more weeks; or (11) other circumstances that the investigators believed compromised participant safety.

During the initial screening visit, a trained clinician assessed participants for a current AUD diagnosis and exclusionary diagnoses using the Structured Clinical Interview for *DSM-5* (SCID-5). Eligible participants then met with the study physician to review medical history, vital signs, electrocardiogram results, and laboratory tests to ensure medical safety for ibudilast treatment.

### Interventions

Randomization was conducted in a 1:1 ratio with participants assigned to either ibudilast or placebo. This allocation was carried out using a stratified block randomization procedure with gender and heavy drinking. MediciNova supplied the medication and matched placebo. The study drug was IBUD and the formulation consisted of 10 mg delayed-release capsules of a generic ibudilast product (Taisho Pharmaceuticals). The target dose was 50 mg twice daily (5 × 10 mg capsules twice daily). To minimize adverse effects, all participants started at 20 mg twice daily (BID) for 2 days, increased to 50 mg BID on day 3, and remained at the 50 mg BID dosing until week 12. During the last 3 days of week 12, participants reduced the dose to 20 mg BID. The medication came in blister packaging and compliance was monitored by the study staff using pill count at each visit. Participants completed the computer-delivered Take Control learning module^23^, which provides suggestions for changing drinking habits.

### Assessments

Participants completed measures of medical safety, alcohol use, and individual differences. At each study visit, participants underwent breath alcohol concentration (BrAC) testing, urine drug screening, and pregnancy testing (for female participants). Depressive symptoms were captured at baseline using the Beck Depression Inventory-II (BDI-II).^24^

### Outcome Measures

The primary endpoint of percentage of heavy drinking days was defined as consuming 4 or more drinks for women or 5 or more drinks for men. Preregistered secondary end points were: drinks per day, drinks per drinking day, and percentage of days abstinent. Alcohol use was assessed at baseline and biweekly during weeks 1 through 12.

### Inflammatory Markers

Blood samples were collected at randomization and weeks 4, 8, and 12. C-reactive protein (CRP) levels were determined utilizing the Human CRP Quantikine ELISA (R&D Systems). Plasma levels of tumor necrotic factor (TNF)-α, interleukin (IL)-6, IL-8, IL-10 and interferon (IFN)-γ were evaluated using a multispot assay system (Meso Scale Discovery). Assays were performed following manufacturers’ protocol.

### Statistical Analysis

#### Main Analysis

The trial had sufficient power to detect a medium effect size (f = 0.28) for the difference between groups in the primary outcome (Critical F = 3.94). With 51 participants in each group, the power to detect such an effect was equal to or greater than 80% for a 1-tailed test at an α level of .05. The 1-tailed *P* value was based on the projection of a favorable response to ibudilast, and not expecting iatrogenic effects. The trial had an enrollment target of 132 individuals, which would allow a 93% power to detect a medium effect size (f = 0.25). The recruitment goal was not met due to COVID-19–related disruptions to clinical research.

Analyses were performed using SAS Statistical Software version 9.4 (SAS Institute Inc). The longitudinal analyses were piecewise linear mixed models^25^ consisting of 2 linear trajectories per medication group: the first phase spanned from baseline to the initial 2-week follow-up, and the second spanned the remaining study period. The model for the primary outcome (PHDD) was

*PHDD_ti_* = *(β_0_ + b_0i_) *+ *(β_1_ + b_1i_)(TIME_ti_) *+ *(β_2_ + b_2i_)(TIMEDIF_ti_) *+ *β_3_(MED_i_) *+ *β_4_(TIME_ti_)(MED_i_) *+ *β_5_(TIMEDIF_ti_)(MED_i_) *+ *ε_ti_*

where *PHDD_ti_* is outcome score at occasion *t* for individual *i*, *MED_i_* is a binary dummy code (0 = placebo, 1 = ibudilast), *TIME_ti_* is a temporal factor that codes the 7 repeated measurements as integers from –1 to 5, and *TIMEDIF_ti_* is a second temporal factor that codes the measurements as 0, 1, 2, 3, 4, and 5. This coding scheme defines the model parameters as follows: β_0_ and β_3_ as the projected placebo group average at the 2-week follow-up and the group mean difference at that occasion, respectively; β_1_ and β_2_ are the placebo group’s linear trend during the first phase and the change to that linear trend in the second phase, respectively; and β_4_ and β_5_ represent group differences in the 2 change rates. Finally, *b*_0i_, *b*_1i_, and *b*_2i_ are normally distributed random effects that allow change trajectories to vary across individuals. The analysis models for the secondary outcomes had a similar composition. Correction for multiple comparison was not implemented as the multiple drinking outcomes test a common hypothesis^26^ of drinking reduction associated with ibudilast. The analyses assumed a conditionally missing at random process where an individual’s missingness is fully determined by their observed data (ie, treatment assignment, covariates, and outcome scores from previous time points). Detailed study procedures and sensitivity analyses are described in eTable 1 and eFigures 1 through 3 in Supplement 1. Group comparisons on demographic and clinical variables used *t* tests for continuous measures and χ^2^ for dichotomous measures.

#### Inflammatory Marker Analysis

Inflammatory marker levels were not normally distributed and were log-transformed prior to statistical analysis. Inflammatory marker analyses were conducted in a multilevel framework using PROC MIXED, where the effect of medication (ibudilast and placebo), time (baseline, follow-up week 4, 8, and 12) and their interaction were examined. Age, sex, smoking status, body mass index, BDI score, baseline drinks per drinking day, inflammatory and anti-inflammatory medications, medication compliance, and randomization inflammatory marker levels were included as covariates. Due to the COVID-19 pandemic, blood samples were only available for 99 participants at baseline, 73 at week 4, 28 at week 8, and 60 at week 12.

## Results

A total of 102 participants were enrolled and randomized in the study (mean [SD] age, 44.3 [10.8] years; 61 male [59.8%]; 24 Black [23.5%], 32 Hispanic [31.4%], 52 White [51.0%]) (Table 1). Fifty-three participants were randomized to ibudilast (mean age, 42.7 years [range, 21-61 years]; 32 male [60.4%]), and 49 were randomized to placebo (mean age, 45.9 years [range, 19-62 years]; 29 male [59.2%]). Trial retention was 87.3% and of those who completed the trial, 10 participants (5 in each medication group) discontinued the study medication.

**Table 1.** Participant Characteristics by Medication Group and Sex

Variable	Participants, No. %					
	Full sample (n = 102)		Ibudilast (n = 53)		Placebo (n = 49)	
	Male (n = 61)	Female (n = 41)	Male (n = 32)	Female (n = 21)	Male (n = 29)	Female (n = 20)
Age, mean (SD), y	45.4 (10.1)	42.6 (11.7)	43.9 (8.9)	40.9 (11.5)	47.0 (11.2)	44.4 (12.1)
Race						
Black or African American	16 (26.2)	8 (19.5)	9 (28.1)	4 (19.1)	7 (24.1)	4 (20.0)
White	24 (39.3)	28 (68.3)	11 (34.4)	14 (66.7)	13 (44.8)	14 (70.0)
≥1 race	10 (16.4)	3 (7.3)	6 (18.8)	1 (4.8)	4 (13.8)	2 (10.0)
Other	11 (18.0)	2 (4.9)	6 (18.8)	2 (9.5)	5 (17.2)	0
Hispanic or Latina/o ethnicity	22 (36.1)	10 (24.4)	12 (37.5)	4 (19.1)	10 (34.5)	6 (30.0)
Heavy drinking days^a^	69.6 (33.7)	62.1 (35.8)	71.1 (35.4)	62.0 (33.3)	68.1 (32.2)	62.2 (39.1)
Drinking days, mean (SD)^a^	23.26 (7.71)	21.51 (6.87)	24.63 (7.10)	19.05 (6.84)	21.76 (8.20)	24.10 (6.03)
Drinks per drinking day, mean (SD)^a^	8.94 (6.26)	5.47 (2.54)	8.68 (4.75)	5.87 (2.85)	9.21 (7.67)	5.05 (2.17)
Alcohol craving (PACS), mean (SD)	12.75 (6.44)	15.32 (5.47)	13.63 (6.46)	14.14 (4.91)	11.79 (6.39)	16.55 (5.87)
AUDIT Score, mean (SD)	20.70 (7.94)	19.54 (6.39)	22.13 (8.78)	19.86 (7.35)	19.14 (6.70)	19.20 (5.38)
Smokes cigarettes	20 (32.8)	20 (48.8)	12 (37.5)	8 (38.1)	8 (27.6)	12 (60.0)
Positive THC screen	15 (24.6)	8 (19.5)	7 (21.9)	5 (23.8)	8 (27.6)	3 (15.0)
BDI-II, mean (SD)	12.66 (8.20)	9.49 (7.07)	13.22 (8.82)	10.10 (7.97)	12.03 (7.58)	8.85 (6.12)
BAI, mean (SD)	7.66 (8.31)	7.93 (6.22)	8.69 (8.74)	8.29 (6.51)	6.52 (7.79)	7.55 (6.05)

Abbreviations: AUDIT, Alcohol Use Disorders Identification Test; BDI-II, Beck Depression Inventory II; BAI, Beck Anxiety Inventory; PACS, Penn Alcohol Craving Scale; THC, Tetrahydrocannabinol.

^a^ Based on Timeline Follow Back collected in the 30 days prior to baseline visit.

Medication compliance was computed using pill count methods and a participant was deemed compliant if they took all the prescribed medication. For the ibudilast group, 28 participants (52.8%) were deemed compliant, 18 (34.0%) were noncompliant, and 7 (13.2%) were missing data. For the placebo group, 19 participants (38.8%) were deemed compliant, 20 (40.8%) were noncompliant, and 10 (20.4%) were missing data. There was no significant difference in compliance between the 2 medication groups (*χ^2^*_1_ = 1.76; *P* = .18). Additionally, there was no group difference in the number of pills remaining (calculated via pill count) between ibudilast (mean [SD] pills, 113.61 [139.76]) and placebo (71.40 [88.35]) groups (*t* = 1.13; *P* = .27).

### Adverse Events

Two participants in the trial experienced serious adverse events, both of which were deemed unrelated to study medication. Similar numbers of participants in the medication groups reported experiencing at least one adverse event during the trial: 36 (67.9%) in the ibudilast group and 27 (55.1%) in the placebo group (χ^2^_1_ = 1.77; *P* = .18). There were no differences between the number of events reported between ibudilast (mean [SD] adverse events, 1.91 [1.14]) and placebo groups (2.23 [1.66]) (*t* = 0.88; *P* = .38). Of the adverse events reported, 91.0% were rated as mild, 5.8% were rated as moderate, and 3.2% were rated as severe and there were no group differences on AE severity (χ^2^_2_ = 0.62; *P* = .73). The most common AE was pain and discomfort, followed by headache and nausea (Table 2).

**Table 2.** Counts and Comparisons for Adverse Events

Adverse event	Overall sample	Ibudilast	Placebo	χ^2^	*P* value
Pain and discomfort	20	11	9	0.07	.79
Headache	16	8	8	0.48	.49
Nausea	16	8	8	0.48	.49
Vomiting	8	5	3	0.10	.75
Flu-like symptoms	8	4	4	0.21	.65
Anxiety	8	4	4	0.21	.65
Insomnia	7	4	3	0.00	.99
Diarrhea	6	4	2	0.24	.63
Dyspepsia	5	3	2	0.02	.90
Fatigue	5	4	1	1.14	.29
Depression	4	2	2	0.10	.76
Eye disorders	3	1	2	0.75	.39
Rash	3	0	3	4.25	.04
Sweating	3	1	2	0.75	.39
Dizziness	2	2	0	1.54	.22
Dysmenorrhea	2	1	1	0.05	.83

### Primary Outcome

The distribution of the PHDD variable was both bounded and bimodal, with substantial proportions of zeros and ones. Adopting a model with normally distributed errors (the *ε_ti_* term in the earlier equation) could yield projected values outside the range of the data. To mitigate this concern, we applied a logit transformation that maps the proportions across the entire number line,^27^ thus eliminating the boundary issue. Analyzing the transformed variable produced effects that were identical to those based on proportions. Given the stability of the findings, we report the PHDD results on the proportion metric.

**Figure 2.**
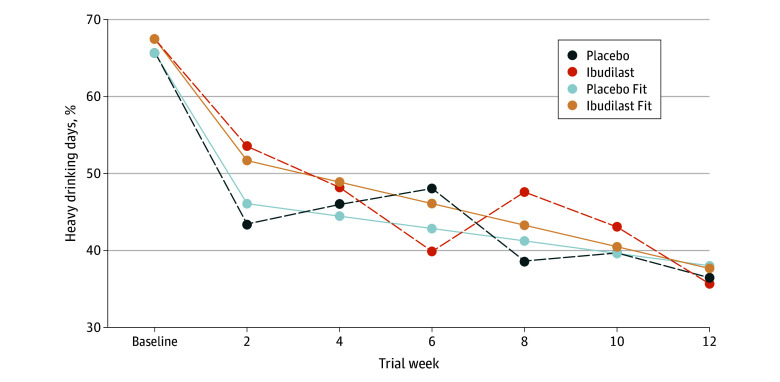
Estimated Percentage of Heavy Drinking Days (PHDD) for Ibudilast and Placebo

The placebo group’s mean significantly decreased by 0.20 during the first 2 weeks (β_1_ = –0.20; 95% CI, –0.30 to –0.09), and the ibudilast group’s biweekly change rate during the same period was not statistically different (β_4_ = 0.04; 95% CI, –0.11 to 0.19]) (Figure 2). During the first 2-week follow-up, the model-projected placebo group average was 0.46 (β_0_ = 0.46; 95% CI, 0.35 to 0.57). The ibudilast group mean PHDD was slightly higher but not statistically different (β_3_ = 0.06; 95% CI –0.10 to 0.21). Expressed as a standardized mean difference, the groups diverged by 0.23 standard deviation units (*d* = 0.23; 95% CI, −0.38 to 0.83), which is consistent with Cohen’s^28^ benchmark for a small effect size.

During the second time period (weeks 4 through 12), the linear change trajectories flattened after the 2-week follow-up (Figure 2B). The placebo group’s biweekly change rate was significantly less negative during the second period relative to the first (β_2_ = 0.18; 95% CI, 0.06 to 0.30), and the ibudilast group’s net change rate during the second period was not statistically different from that of the placebo group (β_5_ = –0.05; 95% CI, −0.22 to 0.1). In absolute terms, the placebo group’s biweekly change rate during the second period was –0.02 (95% CI, –0.04 to 0.01), and the ibudilast group’s rate was –0.03 (95% CI, –0.05 to –0.005); the former was not significantly different from zero, and the latter was. There was a substantial amount of individual-level variation in the growth curves. On a variance explained metric,^29^ the individual growth trajectories explained approximately 8% of the total variation in the outcome scores. Mean PHDD values for week 12 are included in eTable 2 in Supplement 1.

### Secondary Outcomes

The same data analytic approach was used to test the registered secondary drinking outcomes in this trial (eFigure 4 in Supplement 1). In brief, the results were consistent with the primary outcome analysis for PHDD and did not show an effect of ibudilast over placebo for secondary efficacy outcomes (drinks per day [DPD]: β = 0.42, SE = 0.54 [95% CI, −0.65 to 1.48]; *P* = .44; drinks per drinking day [DPDD]: β = 0.79, SE = 0.73 [95% CI, −0.64 to 2.22]; *P* = .28; PDA: β = 0.01, SE = 0.07 [95% CI, −0.13 to 0.14]; *P* = .94) (eTable 2 in Supplement 1).

### Moderation by Depressive Symptomology

Baseline depressive symptoms did not moderate the effects of medication on PHDD (β = 0.001, SE = 0.01 [95% CI, −0.02 to 0.02]; *P* = .95) or DPD (β = −0.06, SE = 0.07 [95% CI, −0.20 to 0.09]; *P* = .45). However, there was a significant BDI × medication × time 1 interaction (β = −0.25, SE = 0.11 [95% CI, −0.47 to −0.03]; *P* = .03) and a significant BDI × medication × time 2 interaction (β = 0.25, SE = 0.11 [95% CI, 0.03 to 0.48]; *P* = .03) for DPDD (eFigure 5 in Supplement 1). There was a significant BDI × medication × time 2 interaction (β = 0.02, SE = 0.01 [95% CI, 0.00 to 0.03]; *P* = .05) for PDA. These findings suggest that at high levels of baseline depressive symptomology, there was a sharper decline in DPDD and PDA in the placebo condition, compared with ibudilast.

### Inflammatory Markers

**Figure 3.**
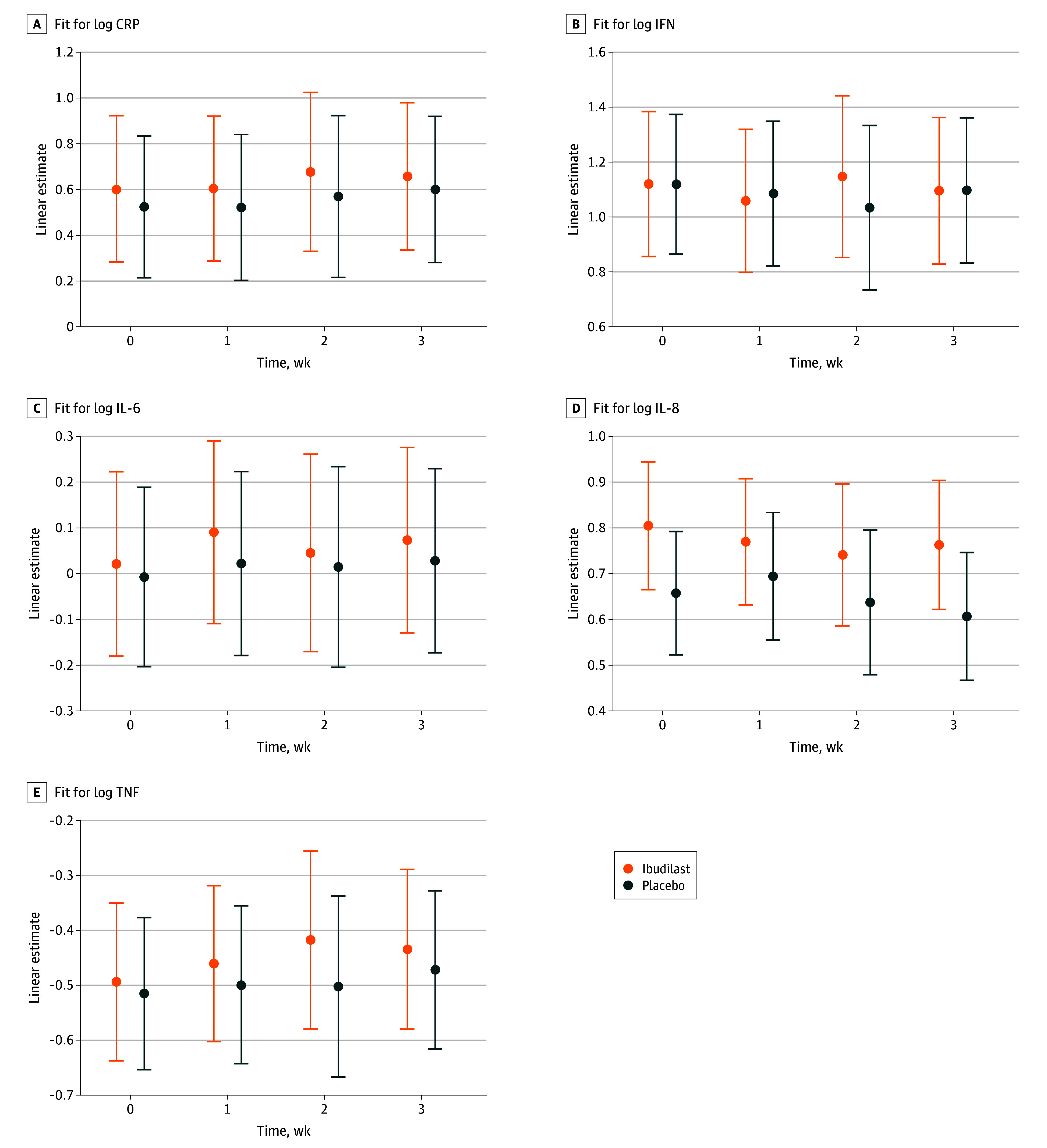
Estimated Inflammatory Markers for Ibudilast and Placebo CRP indicates C-reactive protein; IFN, interferon; IL, interleukin; TNF, tumor necrotic factor.

There was no evidence that ibudilast reduced peripheral markers of inflammation across the 12-week trial, as evidenced by nonsignificant medication main effects on CRP (β = 0.08, SE = 0.10, *t* = 0.82; *P* = .42) (Figure 3A); IFN-γ (β = 0.02, SE = 0.08, *t* = 0.28; *P* = .78) (Figure 3B); IL-6 (β = 0.02, SE = 0.07, *t* = 0.66; *P* = .51) (Figure 3C); IL-8 (β = 0.04, SE = 0.04, *t* = 1.07; *P* = .29) (Figure 3D); IL-10 (β = 0.05, SE = 0.04, *t* = 1.10; *P* = .27) (Figure 3E); or TNF-α (β = −0.02, SE = 0.03, *t* = −0.84; *P* = .40) (Figure 3F). There was no significant medication × time point interaction for the inflammatory markers.

### Moderation by Sex

Exploratory analyses revealed a significant sex × medication interaction (β = −2.48, SE = 1.07 [95% CI, −4.59 to −0.37]; *P* = .02) on drinks per day (eFigure 6 in Supplement 1). The effects of ibudilast vs placebo for reducing drinks per day were stronger in female compared with male participants. A similar result, albeit at not significant, was found for the secondary outcome of percentage of days abstinent (sex × medication interaction: β = 0.26, SE = 0.14 [95% CI, −0.01 to 0.54]; *P* = .06) whereby female participants had an increased percentage of days abstinent compared with male participants.

## Discussion

To our knowledge, this clinical trial is the first to test a neuroimmune modulator for the treatment of AUD. The results did not support the efficacy of ibudilast across all participants in the trial, neither for the primary drinking outcome or any secondary drinking outcomes. Additionally, the results revealed no significant effects of ibudilast on peripheral inflammation marker levels, such that the expected target engagement on inflammatory markers was not observed in this study. Regarding baseline depression as a moderator, results suggested a more favorable placebo response, compared with ibudilast, at high baseline depressive symptomology. Exploratory analyses identified a sex-dependent effect for drinks per day, whereby ibudilast reduced drinks per day more than placebo in female compared with male participants. This finding was in the medium-to-large effect size range and suggests that future studies of ibudilast for female individuals with AUD are warranted. This is noteworthy given the epidemiological data suggesting that women are “closing the gender gap” on AUD prevalence.^30,31,32^

A strong placebo response was observed in this trial, which is consistent with placebo effects reported in other RCTs for AUD.^33,34^ The present study administered Take Control, a modular online program found to reduce alcohol use at similar rates to face-to-face behavioral interventions.^23^ Studies without a behavioral platform and possibly with a longer duration of treatment may be necessary to minimize the placebo effect.

Medication compliance represents another critical factor in elucidating true pharmacological effects in clinical trials. In this study, ibudilast was well-tolerated medication and compliance rates were in the moderate range. The ibudilast formulation required participants to take 5 pills, twice daily, which is not unlike other pharmacotherapies for AUD, such as gabapentin, which is taken via 3 pills, three times daily.^35^ The high pill intake for ibudilast may pose a barrier to compliance and alternative medication delivery methods should be considered for ibudilast. Furthermore, compounds with similar mechanisms of action, such as apremilast,^36^ are currently in development and may provide crucial insights into neuroimmune modulators for AUD. Lastly, precision medicine approaches for AUD pharmacotherapy have identified subgroups of responders for medications such as naltrexone,^37^ gabapentin,^38^ and baclofen.^39^ Hence such efforts are warranted in future studies of neuroimmune modulators.

### Strengths and Limitations

On balance, a number of study strengths and limitations should be considered. Study strengths include a high rate of retention, an online behavioral platform, and stratified randomization. Study limitations include the moderate levels of medication compliance and the influence of COVID-19–related disruptions. It is plausible that some of the clinical effects of ibudilast may have been obscured by COVID-19–related changes in alcohol use. Another limitation of was the lack of biochemical verification for alcohol use and medication adherence. The small sample size and the 1-tailed *P* value also represent limitations.

## Conclusions

This clinical trial found no support for the efficacy of ibudilast for AUD. There were no significant effects of ibudilast on peripheral markers of inflammation. Baseline depressive symptomology was associated with a more favorable response to placebo suggesting a possible iatrogenic response. In post hoc analyses, ibudilast showed benefits for female individuals in reducing drinks per day. These findings largely fail to support ibudilast for the treatment of AUD. Alternative neuroimmune agents may yield a favorable clinical response and subgroups of responders may be identified.

## Notes

Supplement 1.
eMethods.
**eTable 1.** Missing Data Sensitivity Analysis for Percent Heavy Drinking Days Outcome **eTable 2.** Mean Values for Primary, Secondary, and Exploratory Drinking Outcomes for Week 12 **eFigure 1.** Conditionally Missing at Random (Default) **eFigure 2.** Shared Parameter Missing Not at Random (Trajectories Predicting Missingness) **eFigure 3.** Diggle-Kenward Missing Not at Random (Time-Specific PHDD Predicting Missingness) **eFigure 4.** Estimated Means for Secondary Drinking Outcomes at Baseline and Across the Trial for Both IBUD and PLAC Conditions **eFigure 5.** Estimated Means for Drinks per Drinking Day (DPDD) at Baseline and Across the Trial for Both IBUD and PLAC Conditions at Average Levels of Depressive Symptoms **eFigure 6.** Estimated Means for Drinks per Day (DPD) at Baseline and Across the Trial for Both IBUD and PLAC Conditions

Supplement 2.
Trial Protocol

Supplement 3.
Data Sharing Statement
