# Supplementary material for: A Neuroimmune Modulator for Alcohol Use Disorder: A Randomized Clinical Trial
Source: JAMA Netw Open. 2025 Apr 30;8(4):e257523. doi: 10.1001/jamanetworkopen.2025.7523 (PMC12044506; doi:10.1001/jamanetworkopen.2025.7523)
Supplement: Supplement 1. — eMethods. eTable 1. Missing Data Sensitivity Analysis for Percent Heavy Drinking Days Outcome eTable 2. Mean Values for Primary, Secondary, and Exploratory Drinking Outcomes for Week 12 eFigure 1. Conditionally Missing at Random (Default) eFigure 2. Shared Parameter Missing Not at Random (Trajectories Predicting Missingness) eFigure 3. Diggle-Kenward Missing Not at Random (Time-Specific PHDD Predicting Missingness) eFigure 4. Estimated Means for Secondary Drinking Outcomes at Baseline and Across the Trial for Both IBUD and PLAC Conditions eFigure 5. Estimated Means for Drinks per Drinking Day (DPDD) at Baseline and Across the Trial for Both IBUD and PLAC Conditions at Average Levels of Depressive Symptoms eFigure 6. Estimated Means for Drinks per Day (DPD) at Baseline and Across the Trial for Both IBUD and PLAC Conditions [file jamanetwopen-e257523-s001.pdf]

Supplemental Online Content

Ray LA, Meredith LR, Grodin EN, et al. A neuroimmune modulator for alcohol use disorder: a randomized clinical trial. *JAMA Netw Open.* 2025;8(4):e257523.
doi:10.1001/jamanetworkopen.2025.7523

eMethods.

eTable 1. Missing Data Sensitivity Analysis for Percent Heavy Drinking Days Outcome

eTable 2. Mean values for primary, secondary and exploratory drinking outcomes for Week 12

eFigure 1. Conditionally Missing at Random (Default)

eFigure 2. Shared Parameter Missing Not at Random (Trajectories Predicting Missingness)

eFigure 3. Diggle-Kenward Missing Not at Random (Time-Specific PHDD Predicting Missingness)

eFigure 4. Estimated Means for Secondary Drinking Outcomes at Baseline and Across the Trial for Both IBUD and PLAC Conditions

eFigure 5. Estimated Means for Drinks per Drinking Day (DPDD) at Baseline and Across the Trial for Both IBUD and PLAC Conditions at Average Levels of Depressive Symptoms

eFigure 6. Estimated means for drinks per day (DPD) at baseline and across the trial for both IBUD and PLAC conditions

This supplemental material has been provided by the authors to give readers additional information about their work.

© 2025 Ray LA et al. *JAMA Network Open*.

eMethods.

Detailed Study Procedures

Randomization was conducted in a 1:1 ratio with participants assigned to either IBUD or placebo. This allocation was carried out using a stratified block randomization procedure with gender and heavy drinking as the stratification factors. Moderate drinking was defined as ≥ 14 drinks/week for men and ≥ 7 drinks/week for women, while very heavy drinking was defined as ≥ 28 drinks/week for men and ≥ 21 drinks/week for women. The allocation sequence was generated by a computer and executed by the study statistician, who was not involved in participant enrollment. A blinded stratification list was used to assign participants a scenario number and sequence number based on gender and drinking status as stratification criteria. The pharmacist matched this information to the unblinded stratification list and dispensed the prescription. Only the pharmacist had access to the unblinded stratification list. Both the experimental medication and matched placebo were presented as identical white capsules and packaged in blister packs.

The allocation sequence was generated prior to starting the study. Using this sequence, the pharmacist designated IBUD and placebo as either A or B. Only the pharmacist possessed knowledge of whether A or B corresponded to the active medication or the placebo. Subsequently, the laboratory manager blinded this list by transferring it into a blank document, which included scenario and sequence numbers.

Following the medication screening visit, the study physician assessed medical eligibility for participation. If participants were deemed eligible and were interested in participating, they were randomly assigned as described above in a 1:1 ratio to either IBUD or placebo using a stratified block randomization procedure with gender and drinking status as stratification factors. Participants were then matched to a sequence and scenario number based on the blinded stratification list. These numbers were included on the prescription provided to the pharmacist. The pharmacist used the sequence and scenario number in conjunction with the unblinded stratification list to dispense either the active medication or the placebo. Eligible participants were scheduled to pick up their prescriptions. Once participants received their prescriptions, the study coordinator enrolled them in the trial using OnCore, a clinical trial management system.

MediciNova supplied the medication and matched placebo. The study drug was IBUD (MN-166, previously known as AV411), and the formulation consisted of 10-mg delayed-release Pinatos® capsules, the Japanese generic IBUD product produced by Taisho Pharmaceuticals and imported by MediciNova. The target dose was 50 mg BID (5 × 10 mg capsules twice daily). To minimize nausea, IBUD's most common side effect, all participants started at 20 mg BID for 2 days, increased to 50 mg BID on day 3, and remained at the 50 mg BID dosing until week 12. During the last 3 days of week 12, participants reduced the dose (step-down procedure) to 20 mg BID before stopping the medication at the end of the study. The medication came in blister packaging, with each blister pack containing 2 weeks' worth of medication. At the randomization visit's conclusion, participants received three blister packs of medication, approximately 6 weeks of medication (half of the study medication). Participants received the second half of their medication during the week 4 in-person visit. Both study participants and research staff were blinded to medication conditions. Compliance was monitored by the study staff using the pill count method at each follow-up visit. All participants were required to complete the NIAAA-developed and computer-delivered program "Take Control" during the study [1]. This intervention comprised 11 computerized modules that delivered evidence-based, field-tested information to individuals with alcohol problems and provided suggestions for making changes in their drinking habits.

Detailed Study Measures

To streamline data management, we collected most self-reported study measures using Qualtrics. During the in-person screening visit, the following interviews and self-report measures were administered to assess eligibility and relevant individual differences: (1) Timeline FollowBack (TLFB) interview measured the quantity and frequency of drinking [2]; (2) SCID-5 [3] for current AUD diagnosis and exclusionary diagnoses (e.g., lifetime psychosis); (3) CIWA-AR [4] to assess alcohol withdrawal symptoms; (4) Beck Anxiety Inventory (BAI)[5] to measure anxiety symptomatology; (5) Beck Depression Inventory-II (BDI-II)[6] to capture depressive symptomatology; (6) Penn Alcohol Craving Scale (PACS)[7] to measure alcohol craving; and (7) Alcohol Use Disorders Identification Test (AUDIT)[8] to identify harmful patterns of alcohol consumption. At each study visit participants underwent breath alcohol concentration (BrAC) testing, urine drug screening, and pregnancy testing (for females). A BrAC = 0.00 g/dl was required for participation.

Circulating levels of peripheral C reactive protein (CRP) were measured in plasma. Blood samples were collected by venipuncture into EDTA tubes, placed on ice, centrifuged for acquisition of plasma, and stored at -80°C for batch testing. CRP levels were determined utilizing the high-sensitivity Human CRP Quantikine ELISA (R&D Systems). Assays were performed according to the manufacturer's protocol, with a lower limit of detection of 0.2 mg/L. Samples were assayed in duplicate. Intra- and inter-assay variation of the tests was <5.7%. For the small proportion (8%, n=8) of samples with CRP levels below the limit of detection (0.2 mg/L), a value of 0.2 mg/L was assigned. As expected, CRP

© 2025 Ray LA et al. *JAMA Network Open*.

values were non-normally distributed (skewness=4.90, kurtosis=27.16) and therefore logarithmically (log) transformed for analyses. Participants with a CRP level > 3 mg/L at baseline are classified as high CRP, as CRP levels >3 mg/L predict high-risk for cardiovascular events [9]. This classification was used for the purpose of visualizing the results, while the statistical models used a continuous measure of CRP (log transformed).

Missing Data Sensitivity Analyses

The longitudinal analyses reported in the paper body assumed a conditionally missing at random process where an individual's missingness is fully determined by their observed data (i.e., treatment assignment, covariates, and outcome scores from previous waves). While this is a standard assumption for missing data analyses, it requires the untestable proposition that unseen score values themselves carry no additional information about missingness. To explore this issue, we performed a sensitivity analysis that considered a pair of selection models that invoke a missing not at random (MNAR) assumption where dropout links to *unobserved* drinking levels. The primary outcome for the sensitivity analyses was the percentage of heavy drinking days (PHDD). Selection models augment the focal linear mixed model with an additional probit regression model with a binary dropout indicator as the outcome (0 = measurement occasion prior to dropout, 1 = measurement occasion at dropout). We used MCMC estimation in the Blimp data analysis software for all models [10]. The computational details for Blimp's missing not at random selection models are found in Du, Enders, Keller, Bradbury, Karney [11].

The shared parameter selection model [12] was the first MNAR analysis. As described in the main paper, the focal analysis model featured random intercepts and slopes that allowed growth trajectories to vary across person. The shared parameter model uses the random intercepts and slopes as predictors in the dropout model. This model is useful for situation where a participant's underlying (latent) growth trajectory could be responsible for missing data (e.g., participants experiencing the most rapid declines in drinking might differentially quit compared to those with less improvement). In addition to the random intercepts and slopes, the dropout model also included time dummy codes, medication arm, and time-by-medication arm.

The Diggle–Kenward selection model [13] was the second MNAR analysis. Instead of trajectories influencing dropout, this model links dropout at time t to the unseen PHDD scores at time t and the observed PHDD scores at the previous measurement occasion. This model is useful for situation where time-specific realizations of one's drinking behavior could be responsible for missing data (e.g., participants experiencing a sudden increase in PHDD at time t have differential dropout). In addition to the concurrent and lagged PHDD effects, the dropout model again included time dummy codes, medication arm, and time-by-medication arm.

Table S1 displays the focal mixed model parameter summaries from the default conditionally missing at random analysis and the two MNAR models. To help convey the differences, **Figures S1, S2, and S3** show the predicted growth trajectories. **Figure S1** corresponds to the primary outcome analysis from the main paper, and **Figures S2 and S3** are the supplemental MNAR models. The three analyses exhibited noticeable differences in some parameters. However, the overall patterns of change and the substantive conclusions reported in the main paper were consistent across different analyses.

The results presented in **Table S1** invoke very different assumptions about the dropout processes, any of which could be plausible. It is important to emphasize that models for MNAR processes also require the strict, unverifiable assumption that the missingness model is correctly specified (i.e., has the correct constellation of effects predicting dropout). Following common practice in the literature [14,e.g., 15], Table S5 also presents two information criteria, the DIC2 and WAIC [16]. The conditionally missing at random analysis achieved the best fit (lowest DIC2 and WAIC), followed by the shared parameter model and the Diggle–Kenward model. These indices can sometimes distinguish among competing models if one is willing to accept the validity of the models' predictions about the missing values with no input from the observed data.

References

1. Devine EG, Ryan ML, Falk DE, Fertig JB, Litten RZ. An exploratory evaluation of Take Control: A novel computer-delivered behavioral platform for placebo-controlled pharmacotherapy trials for alcohol use disorder. *Contemp Clin Trials.* 2016;50:178-185.
2. Sobell LC, Sobell MB. Timeline Follow-Back. In: Litten RZ, Allen JP, eds. *Measuring Alcohol Consumption: Psychosocial and Biochemical Methods.* Totowa, NJ: Humana Press; 1992:41-72.
3. First MB, Williams JBW, R.S. K, Spitzer RL. *Structured Clinical Interview for DSM-5—Research Version (SCID-5 for DSM-5, Research Version; SCID-5-RV).* Arlington, VA: American Psychiatric Association; 2015.
4. Sullivan JT, Sykora K, Schneiderman J, Naranjo CA, Sellers EM. Assessment of alcohol withdrawal: the revised clinical institute withdrawal assessment for alcohol scale (CIWA-Ar). *Br J Addict.* 1989;84(11):1353-1357.

© 2025 Ray LA et al. *JAMA Network Open*.

5. Beck AT, Epstein N, Brown G, Steer RA. An inventory for measuring clinical anxiety: psychometric properties. *J Consult Clin Psychol.* 1988;56(6):893-897.
6. Beck AT, Steer RA, Brown GK. *BDI-II, Beck depression inventory : manual.* 1996.
7. Flannery BA, Volpicelli JR, Pettinati HM. Psychometric properties of the Penn Alcohol Craving Scale. *Alcohol Clin Exp Res.* 1999;23(8):1289-1295.
8. Saunders JB, Aasland OG, Babor TF, de la Fuente JR, Grant M. Development of the Alcohol Use Disorders Identification Test (AUDIT): WHO Collaborative Project on Early Detection of Persons with Harmful Alcohol Consumption--II. *Addiction.* 1993;88(6):791-804.
9. Ridker PM. Clinical application of C-reactive protein for cardiovascular disease detection and prevention. *Circulation.* 2003;107(3):363-369.
10. Keller BT, Enders CK. Blimp user's guide (Version 3). 2021. www.appliedmissingdata.com/blimp.
11. Du H, Enders CK, Keller BT, Bradbury T, Karney B. A Bayesian latent variable selection model for nonignorable missingness. *Multivariate Behavioral Research.* 2021:Advance online publication.
12. Albert PS, Follmann DA. Shared-parameter models. In: Fitzmaurice G, Davidian M, Vebeke G, Molenberghs G, eds. *Longitudinal data analysis.* Boca Raton, FL: Chapman & Hall; 2009.
13. Diggle P, Kenward MG. Informative drop-out in longitudinal data analysis. *Journal of the Royal Statistical Society: Series C (Applied Statistics).* 1994;43(1):49–93.
14. Muthén B, Asparouhov T, Hunter AM, Leuchter AF. Growth modeling with nonignorable dropout: Alternative analyses of the STAR*D antidepressant trial. *Psychol Methods.* 2011;16(1):17–33.
15. Ibrahim JG, Chen MH, Lipsitz SR, Herring AH. Missing-data methods for generalized linear models: A comparative review. *Journal of the American Statistical Association.* 2005;100(469):332–346.
16. Du H, Keller B, Alacam E, Enders C. Comparing DIC and WAIC for multilevel models with missing data. *Behavior Research Methods.* 2024;56:2731–2750.

© 2025 Ray LA et al. *JAMA Network Open*.

eTable 1. Missing Data Sensitivity Analysis for Percent Heavy Drinking Days Outcome

Parameter	Est.	SE	2.5% LCL	97.5% UCL	Chi Square	*p*
Conditionally Missing at Random (Default)						
Intercept	0.45	0.06	0.33	0.57	55.25	0.000
Med	0.06	0.08	-0.11	0.22	0.48	0.49
Time1	-0.20	0.06	-0.32	-0.08	10.66	0.001
Time2	0.18	0.07	0.05	0.31	7.42	0.01
Med*Time1	0.04	0.08	-0.12	0.21	0.27	0.60
Med*Time2	-0.06	0.09	-0.23	0.12	0.38	0.54
DIC2	1807.66					
WAIC	2168.84					
Shared Parameter Missing not at Random (Trajectories Predicting Missingness)						
Intercept	0.50	0.07	0.38	0.64	60.31	0.000
Med	0.05	0.09	-0.12	0.22	0.32	0.57
Time1	-0.16	0.06	-0.28	-0.03	6.51	0.01
Time2	0.14	0.07	-0.01	0.27	3.70	0.05
Med*Time1	0.05	0.08	-0.11	0.22	0.38	0.54
Med*Time2	-0.07	0.10	-0.25	0.12	0.47	0.49
DIC2	1868.66					
WAIC	2218.23					
Diggle–Kenward Missing not at Random (Time-Specific PHDD Predicting Missingness)						
Intercept	0.38	0.06	0.26	0.51	35.85	0.000
Med	0.09	0.09	-0.09	0.26	0.96	0.33
Time1	-0.26	0.06	-0.38	-0.14	16.70	0.000
Time2	0.23	0.07	0.10	0.37	11.05	0.001
Med*Time1	0.07	0.09	-0.09	0.25	0.74	0.39
Med*Time2	-0.08	0.09	-0.27	0.09	0.75	0.39
DIC2	2342.94					
WAIC	2724.13					

© 2025 Ray LA et al. *JAMA Network Open*.

eTable 2. *Mean values for primary, secondary and exploratory drinking outcomes for Week 12*

Variable	Ibudilast (n=53)	Placebo (n=49)
%Heavy Drinking Days (SD)	34.97 (38.75)	35.34 (40.07)
Drinks Per Day (SD)	2.26 (2.21)	1.91 (2.09)
Drinks Per Drinking Day (SD)	4.13 (3.18)	3.41 (2.50)
% Days Abstinent (SD)	54.86 (34.42)	55.36 (38.61)

© 2025 Ray LA et al. *JAMA Network Open*.

eFigure 1. Conditionally Missing at Random (Default)

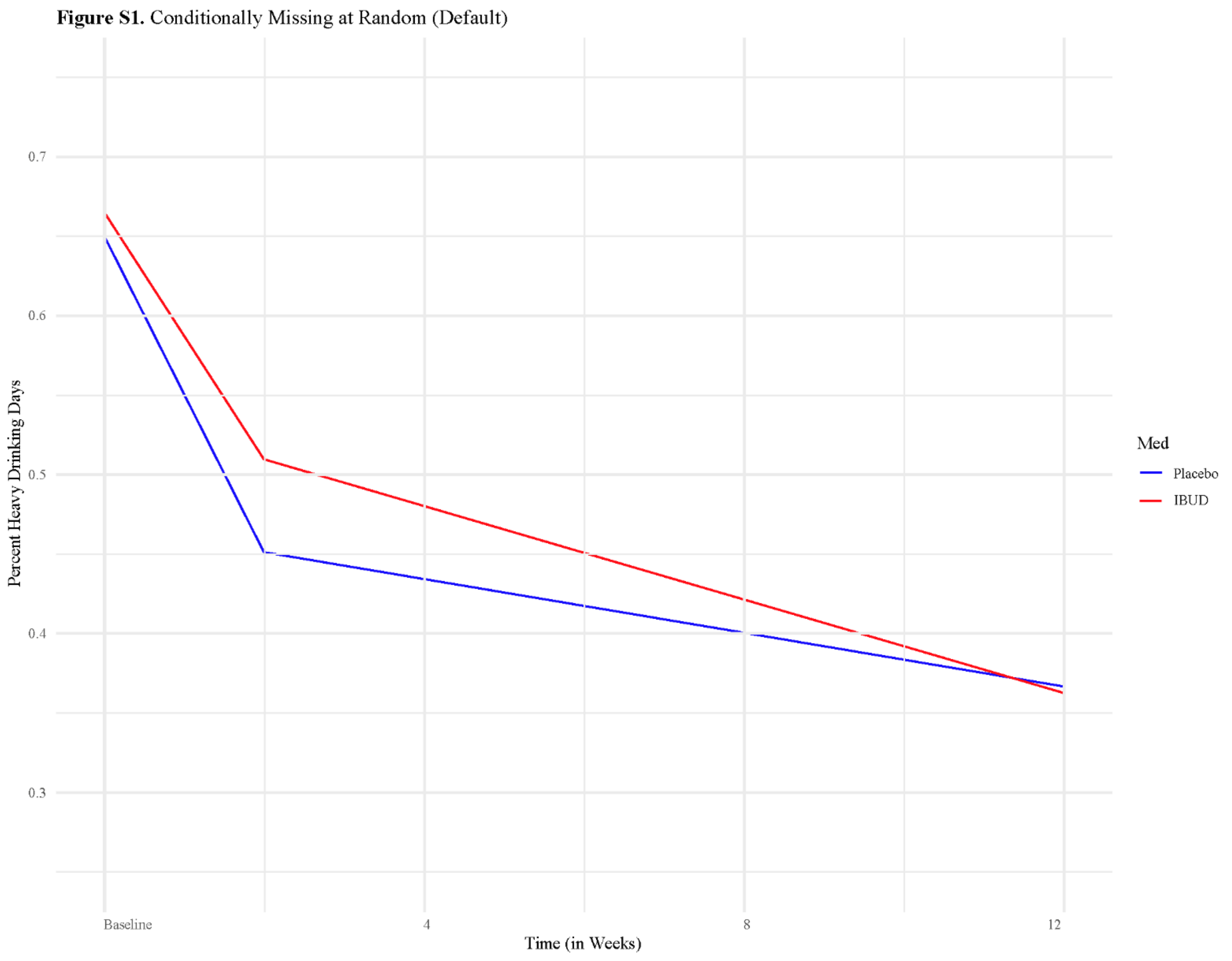

© 2025 Ray LA et al. *JAMA Network Open*.

eFigure 2. Shared Parameter Missing Not at Random (Trajectories Predicting Missingness)

Figure S2. Shared Parameter Missing not at Random (Trajectories Predicting Missingness)

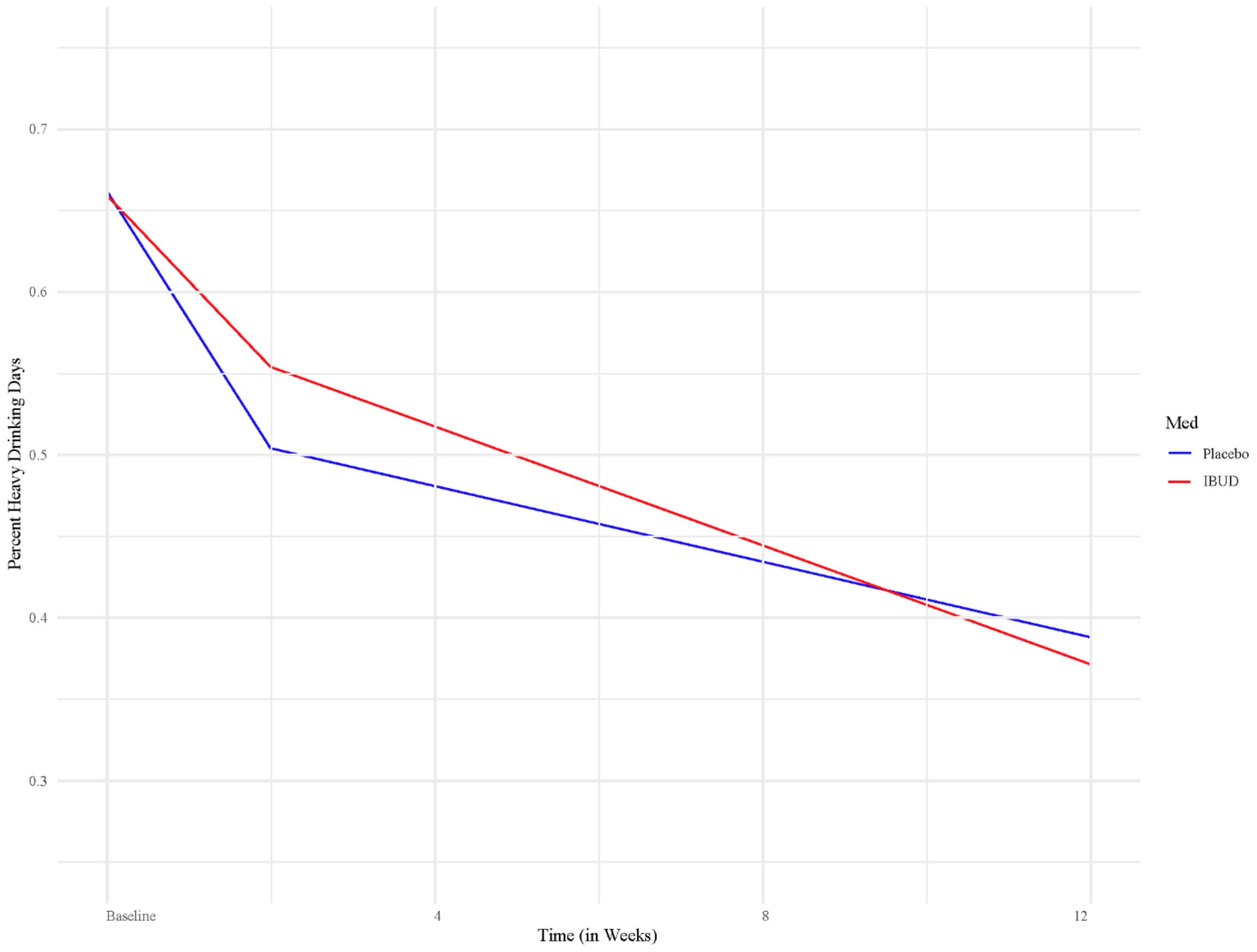

© 2025 Ray LA et al. *JAMA Network Open*.

eFigure 3. Diggle-Kenward Missing Not at Random (Time-Specific PHDD Predicting Missingness)

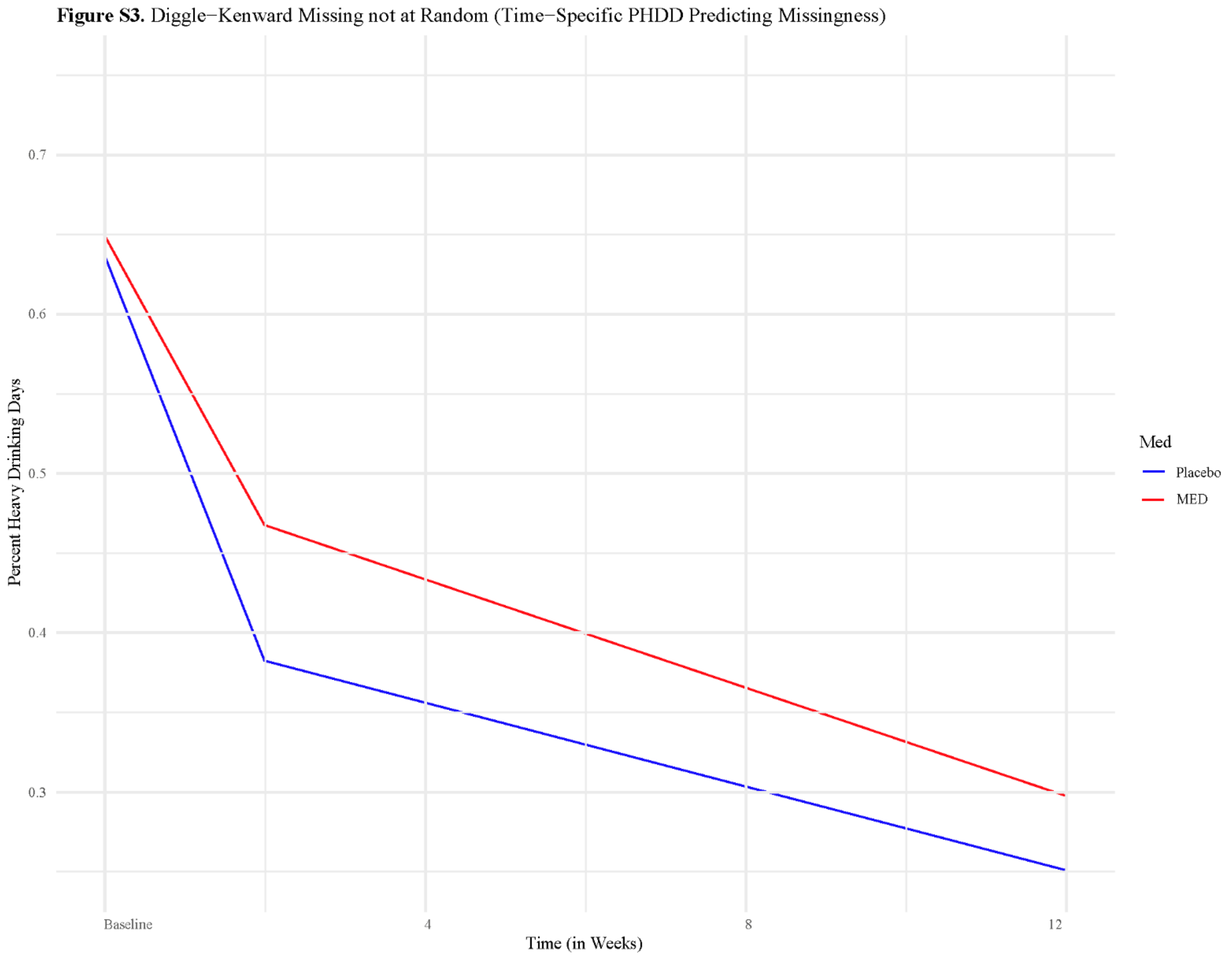

© 2025 Ray LA et al. *JAMA Network Open*.

eFigure 4. Estimated means for secondary drinking outcomes at baseline and across the trial for both IBUD and PLAC conditions

(A) Drinks per day

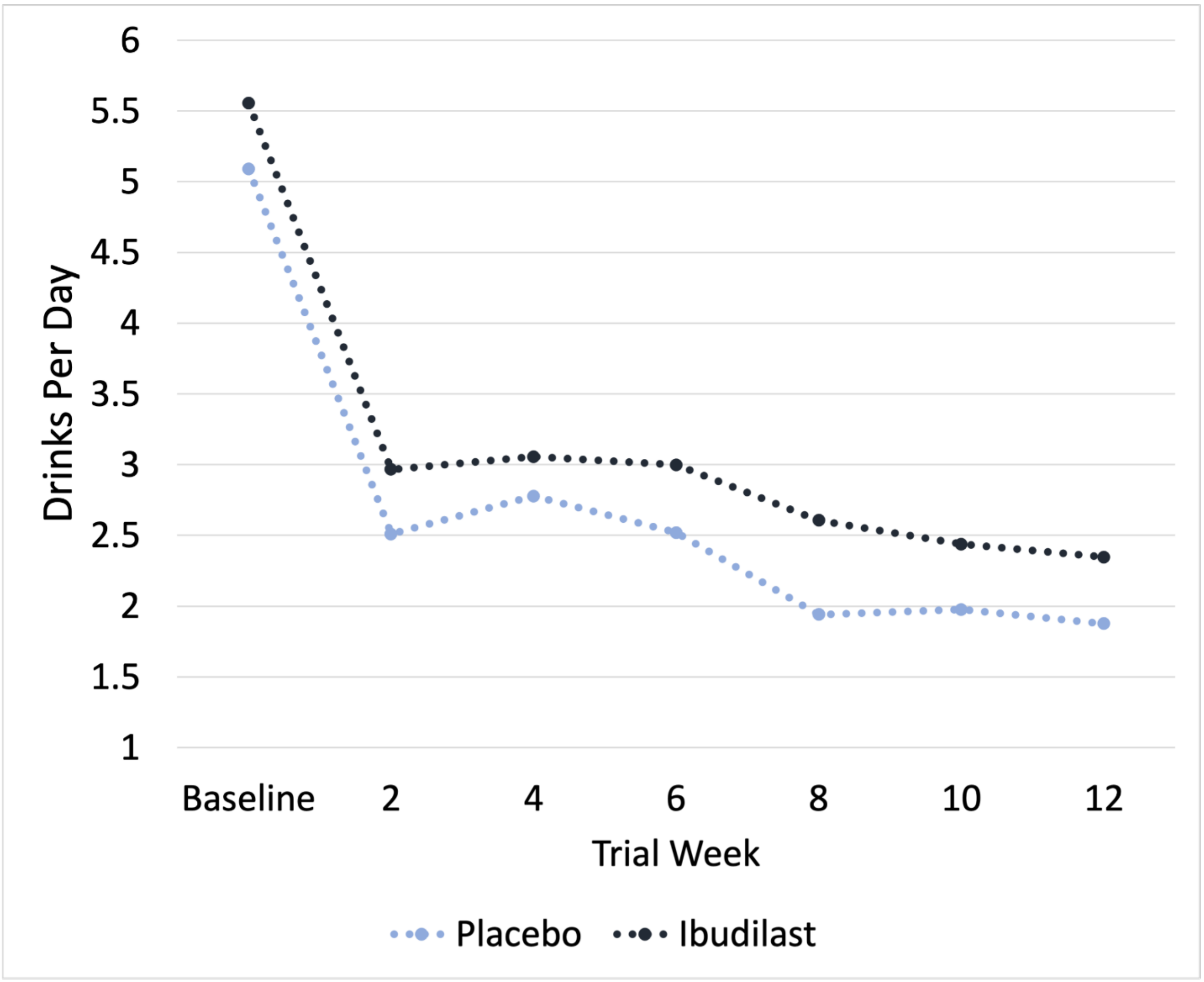

© 2025 Ray LA et al. *JAMA Network Open*.

(B) Drinks per drinking day

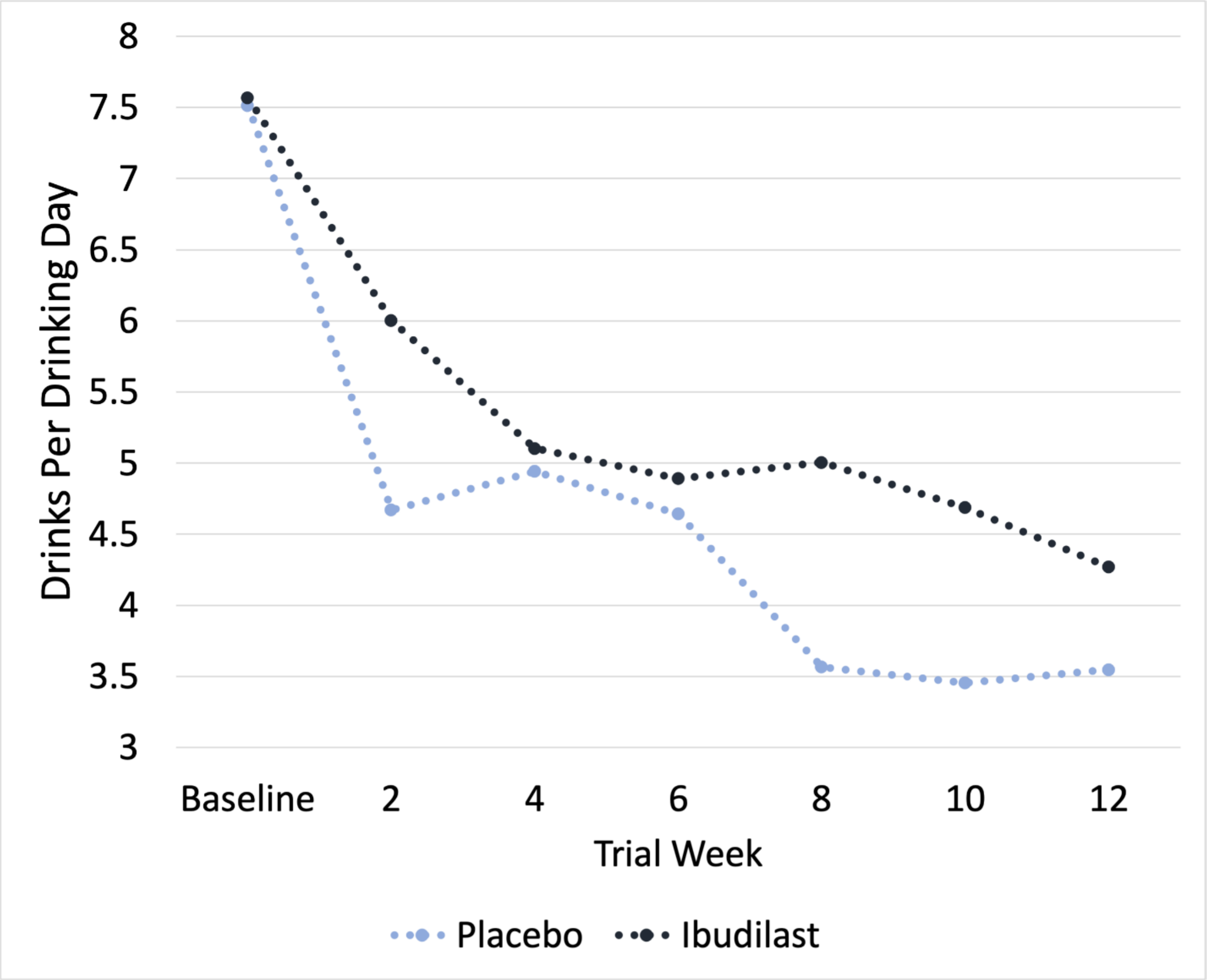

© 2025 Ray LA et al. *JAMA Network Open*.

(C) Percent days abstinent

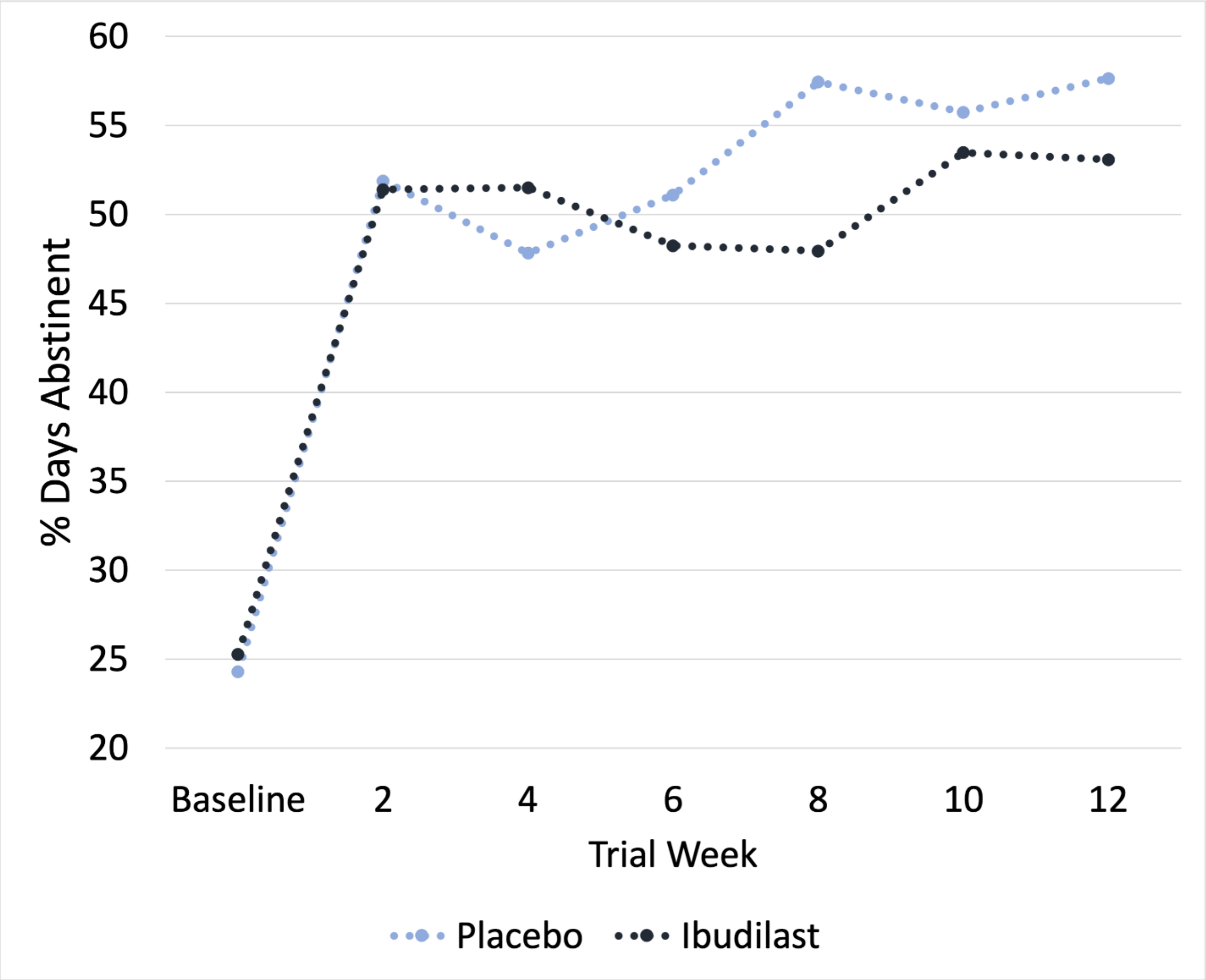

© 2025 Ray LA et al. *JAMA Network Open*.

(D) Percentage of the sample abstinent

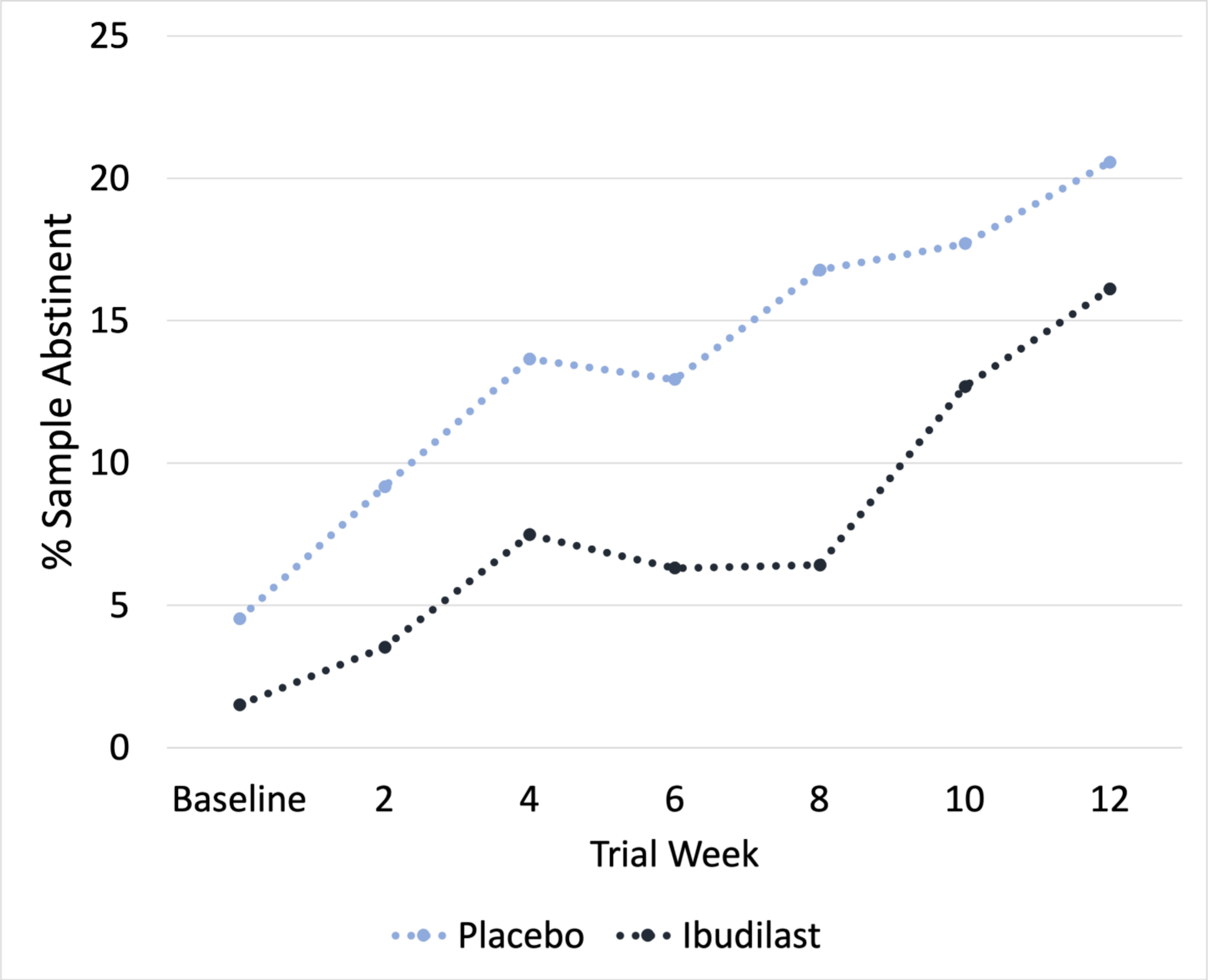

© 2025 Ray LA et al. *JAMA Network Open*.

(E) Percentage of the sample with no heavy drinking

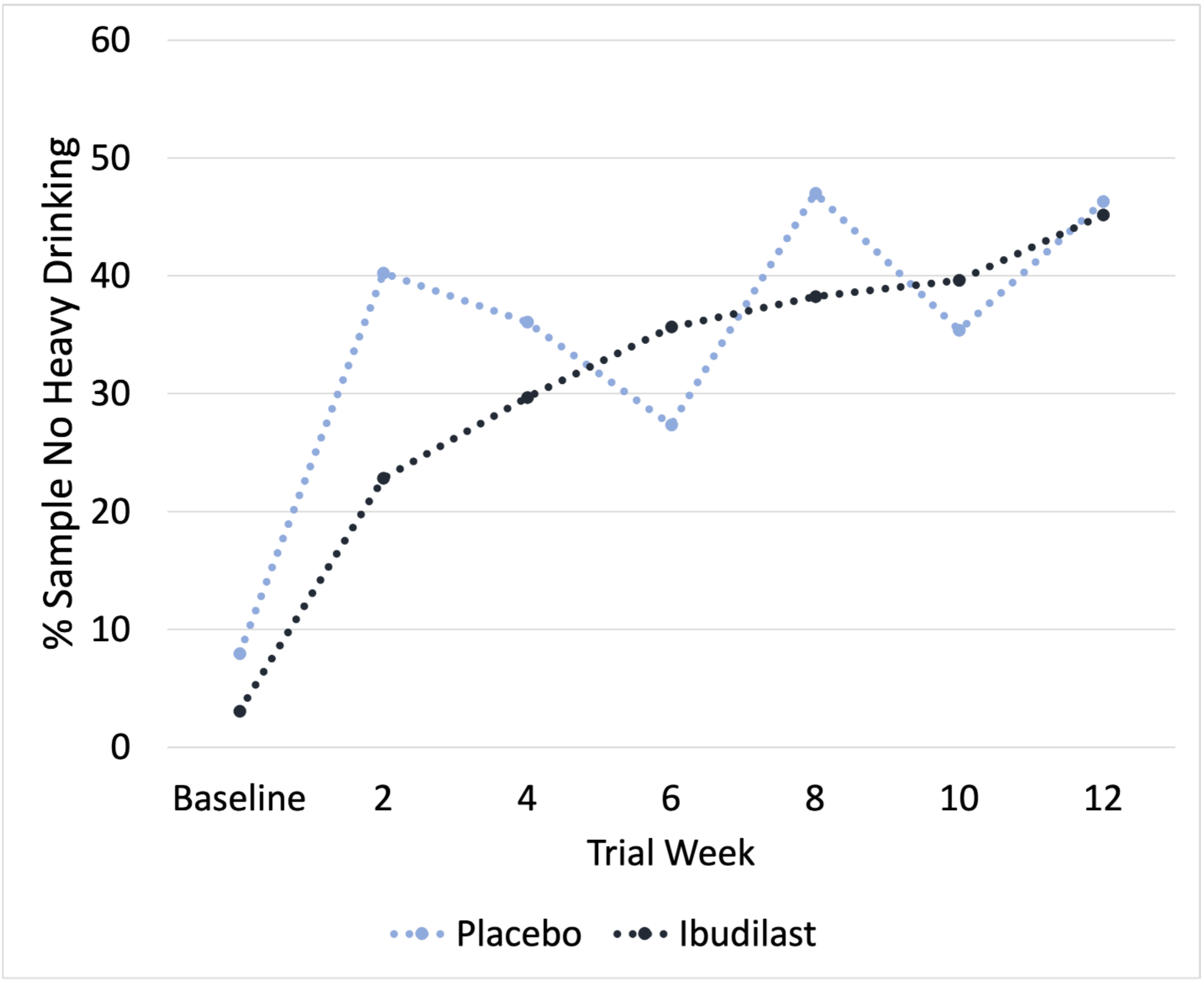

© 2025 Ray LA et al. *JAMA Network Open*.

eFigure 5. Estimated means for drinks per drinking day (DPDD) at baseline and across the trial for both IBUD and PLAC conditions at average levels of depressive symptoms
(**3a**), at high levels of depressive symptoms (one SD above the mean; **3b**), and at low levels of depressive symptoms (one SD below the mean; **3c**). Estimated means for Percent Days Abstinent (PDA) at baseline and across the trial for both IBUD and PLAC conditions at average levels of depressive symptoms (**3d**), at high levels of depressive symptoms (one SD above the mean; **3e**), at low levels of depressive symptoms (one SD below the mean; **3f**).

(A)

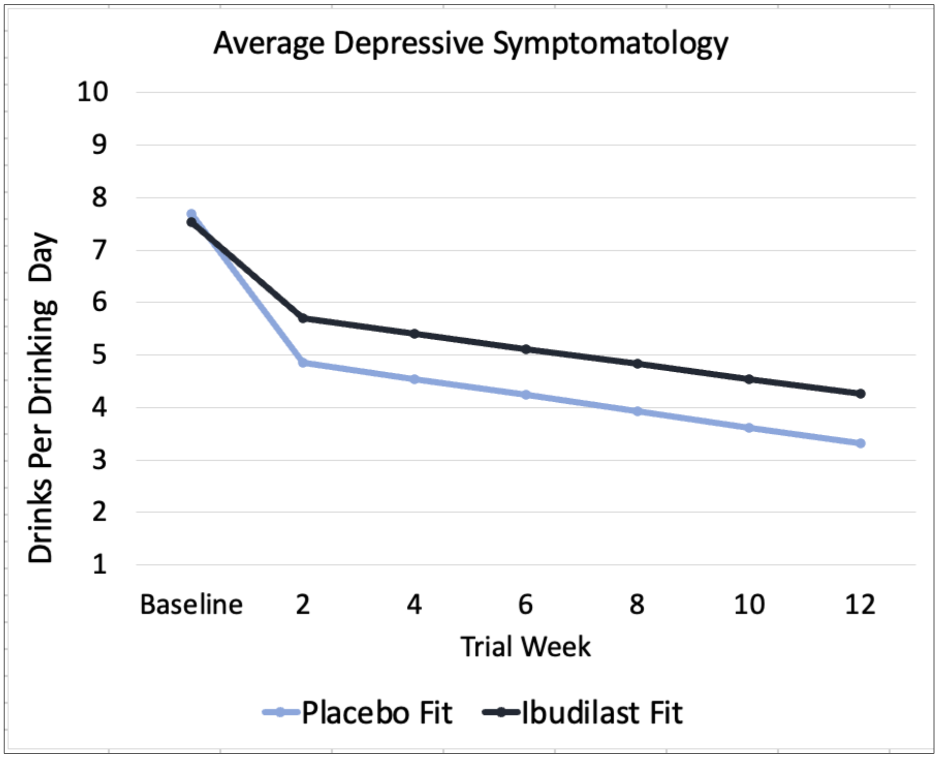

© 2025 Ray LA et al. *JAMA Network Open*.

(B)

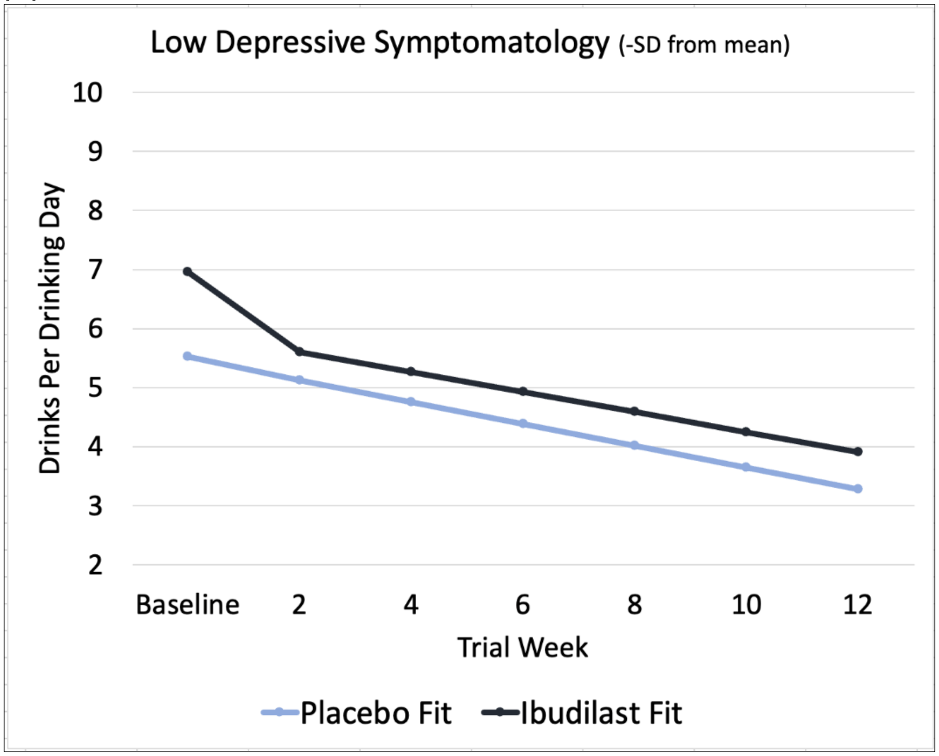

(C)

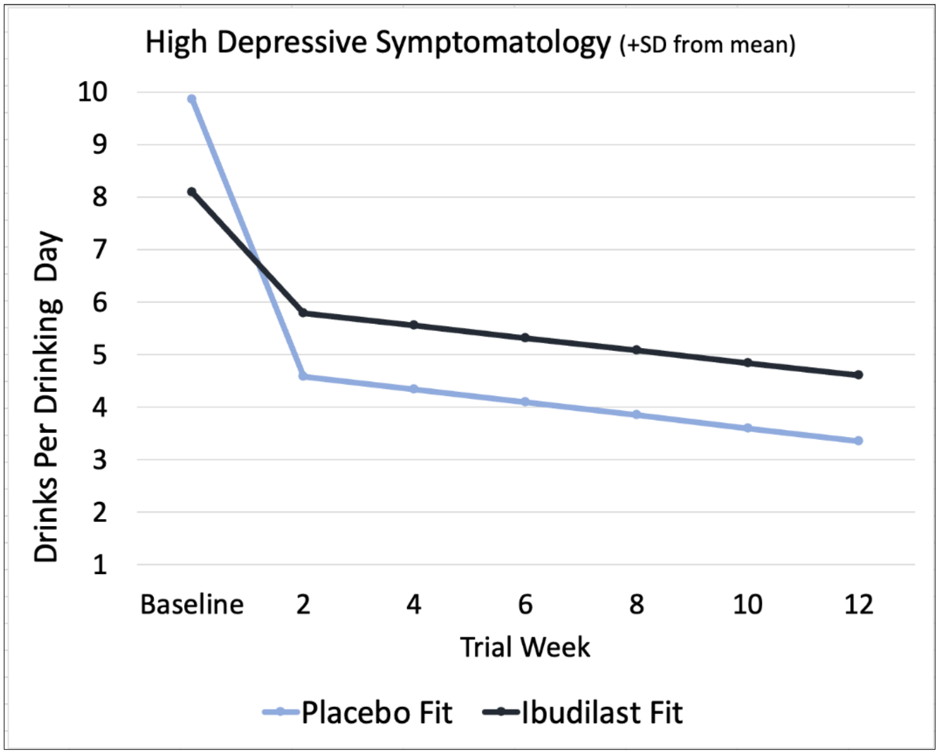

© 2025 Ray LA et al. *JAMA Network Open*.

(D)

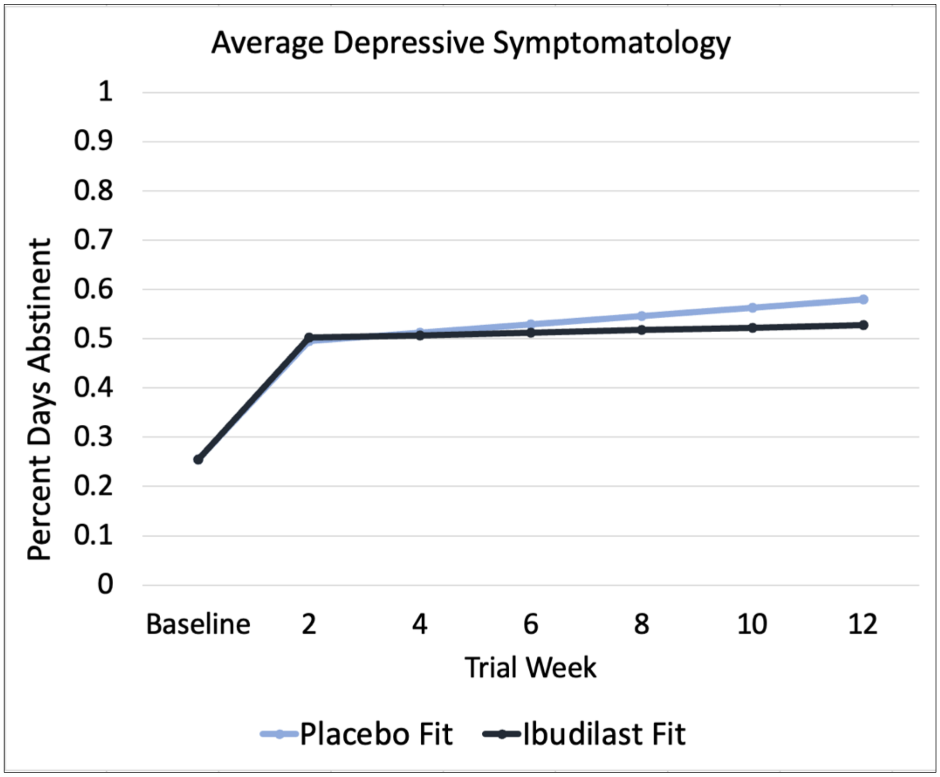

(E)

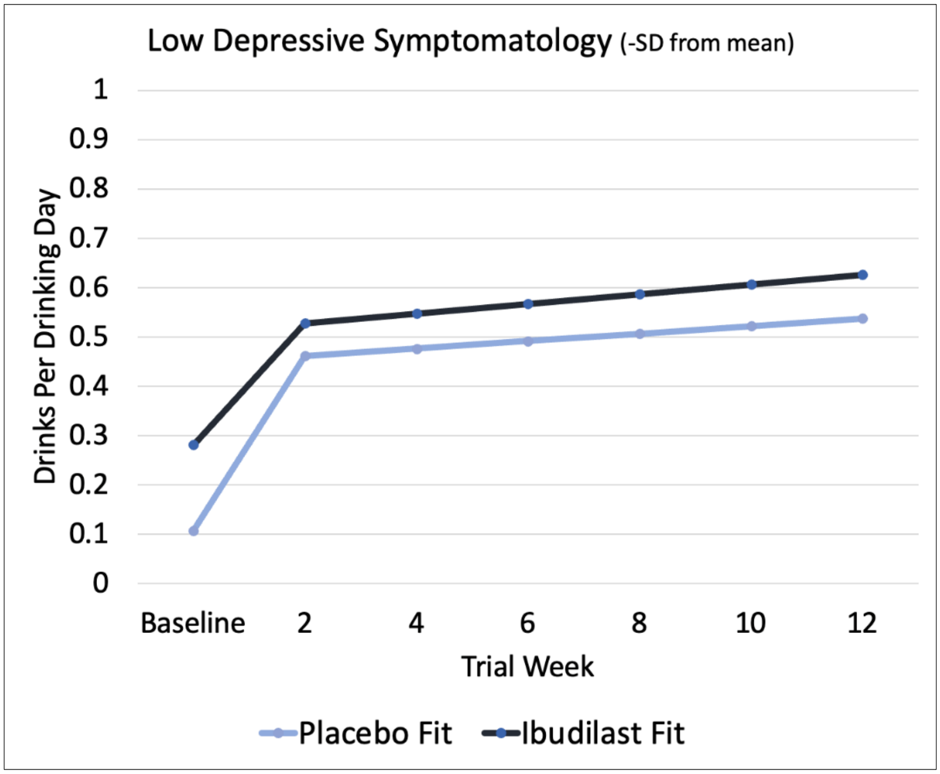

© 2025 Ray LA et al. *JAMA Network Open*.

(F)

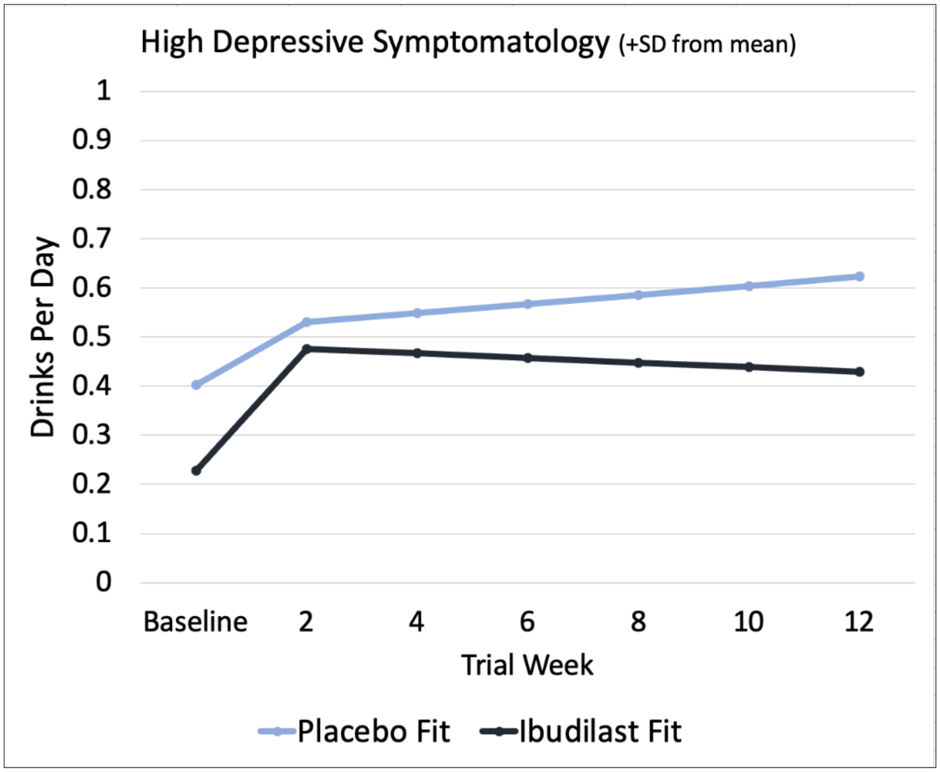

© 2025 Ray LA et al. *JAMA Network Open*.

eFigure 6. Estimated means for drinks per day (DPD) at baseline and across the trial for both IBUD and PLAC conditions for males (**5a**) and females (**5b**).

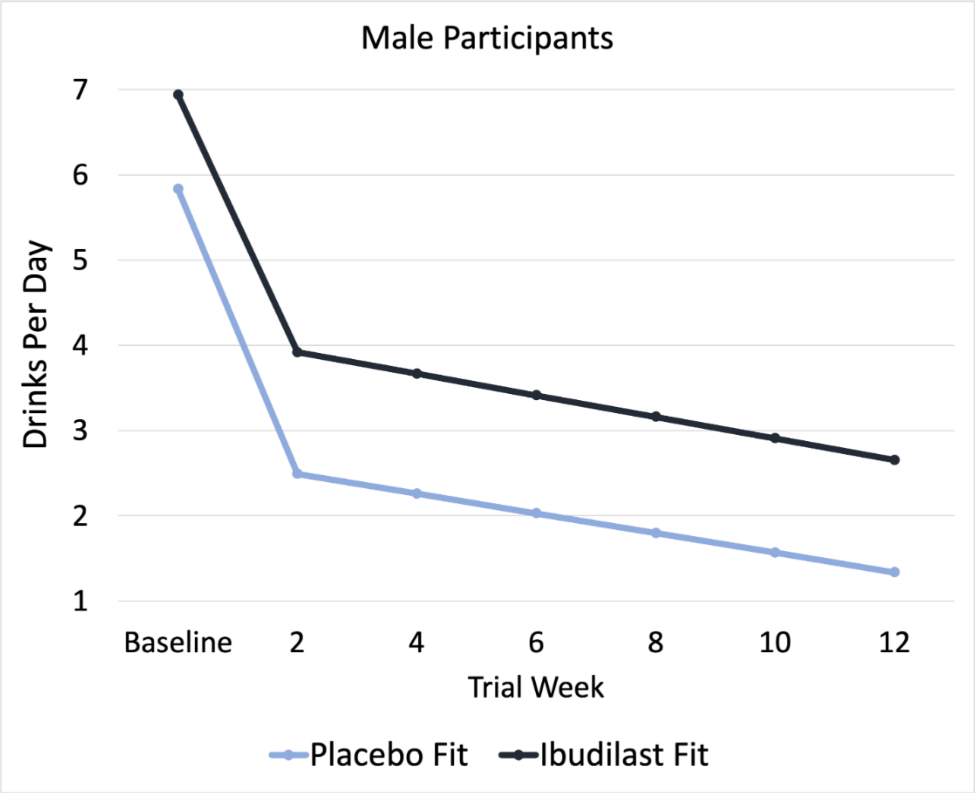

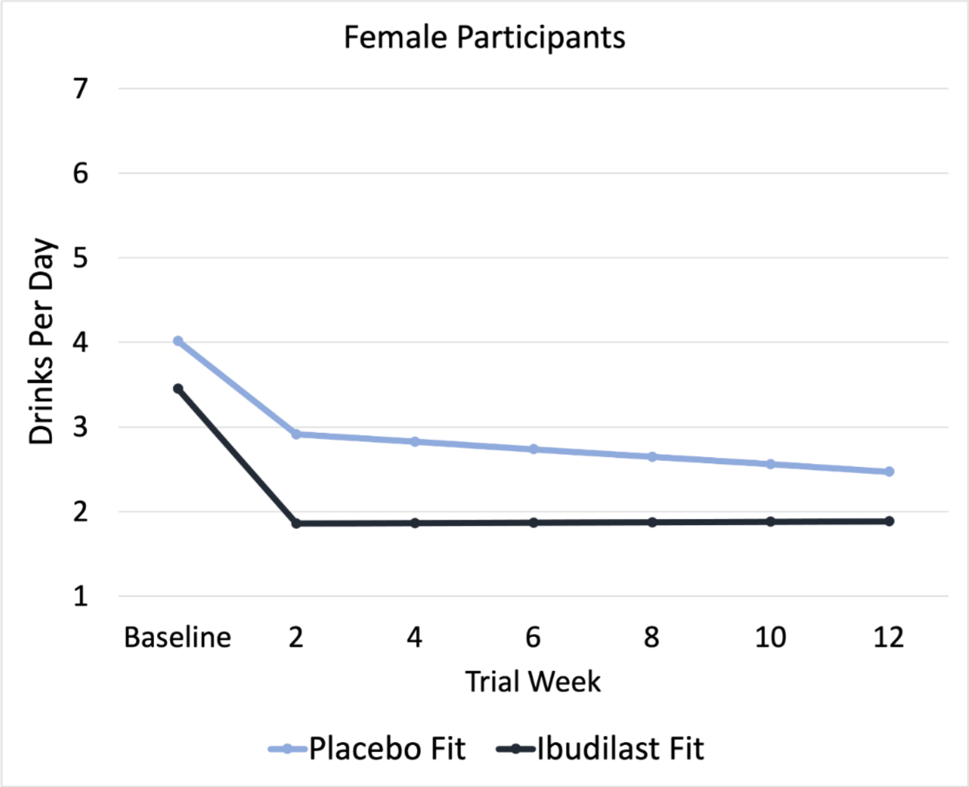

© 2025 Ray LA et al. *JAMA Network Open*.
