# Supplementary material for: A Neuroimmune Modulator for Alcohol Use Disorder: A Randomized Clinical Trial
Source: JAMA Netw Open. 2025 Apr 30;8(4):e257523. doi: 10.1001/jamanetworkopen.2025.7523 (PMC12044506; doi:10.1001/jamanetworkopen.2025.7523)
Supplement: Supplement 3. — Data Sharing Statement [file jamanetwopen-e257523-s003.pdf]

Data Sharing Statement

Ray. A Neuroimmune Modulator for Alcohol Use Disorder. *JAMA Netw Open*. Published April 30, 2025. doi:10.1001/jamanetworkopen.2025.7523

Data
Additional Information: ClinicalTrials.gov NCT03594435
Data available: No

Additional Information
Explanation for why data not available: We are still analyzing the data for secondary publications.
